# Early Infant Feeding Practices as Possible Risk Factors for Immunoglobulin E-Mediated Food Allergies in Kuwait

**DOI:** 10.1155/2018/1701903

**Published:** 2018-06-03

**Authors:** Dalal Alkazemi, Munirah Albeajan, Stan Kubow

**Affiliations:** ^1^Department of Food Science and Nutrition, College of Life Sciences, Kuwait University, Kuwait; ^2^Department of Food and Nutrition, Mubarak Al-Kabeer Hospital, Kuwait; ^3^School of Human Nutrition, McGill University, 21,111 Lakeshore, Ste-Anne-de-Bellevue, QC H9X3V9, Canada

## Abstract

**Objective:**

Early feeding and infant exposures have been suggested as potential risk factors for immunoglobulin E- (IgE-) mediated food allergy (FA). We aimed to evaluate the association between IgE-mediated FA in children and early exposures including the child's nutritional status, breastfeeding and its duration, the age at which the solid food was first introduced, antibiotic exposure during the first year of life, and the child's vitamin D status during infancy.

**Design:**

A case-control study.

**Setting and Subjects:**

Children aged 0–13 years were recruited from pediatric allergy and immunology clinics (PAICs) located at major government hospitals in Kuwait (total FA cases: *n* = 100; boys = 67%), and healthy controls (*n* = 100, boys 55%) were recruited from various vaccination units at primary healthcare centers.

**Results:**

Cow's milk allergy was the most common type of FA. FA status was independently associated with the early exposures of exclusive breastfeeding (aOR = 15.55 (3.26–74.19), *p* = 0.001), vitamin D deficiency or insufficiency during infancy (aOR = 5.42 (1.92–15.30), *p* = 0.001), and antibiotic exposure during the first year of life (aOR = 5.00 (1.58–15.84), *p* = 0.006).

**Conclusions:**

FA is highly prevalent among children in Kuwait, and our data indicate that early nutrition-related and antibiotic exposures are associated with FA risk.

## 1. Introduction

Food allergies (FAs) and other atopic diseases such as asthma, rhinitis, and atopic dermatitis (eczema) are an increasing public health burden, affecting both adults and children [1]. Globally, it is estimated that FAs affect nearly 5% of adults and 8% of children, with growing evidence of an increase in their prevalence [2]. Immunoglobulin E- (IgE-) mediated allergic reactions are most commonly associated with FAs and induce a variety of symptoms that are rapid in onset and directly related to food ingestion [3]. The most common symptoms of IgE-mediated, food-induced allergic reactions include those on the skin as well as gastrointestinal and respiratory symptoms. FA can also affect the growth and nutritional status of children [4]. The mean *z*-scores of both weight and height for age were shown to decrease as the number of sensitized food allergens increased [5].

FA is caused by a complex interplay of environmental exposures, genetic variants, gene-environment interactions, and epigenetic alterations [6]. Epidemiologic studies suggest the importance of environmental influences, which may be derived from factors such as nutrition, the gut microbiome, or a combination of the two [7]. The changes in the intestinal colonization pattern during infancy can also be caused by many other factors such as cesarean delivery, the presence of siblings, and antibiotics use. Hirsch et al. [8] found that the administration of antibiotics in the first year of life was associated with FA in young children, and Love et al. [9] noted that multiple antibiotic prescriptions were strongly associated with increased odds of FA diagnosis.

Recently conducted studies show that prenatal and infant feeding are key environmental exposures that play a fundamental role in the maturation process of the immune system and in shaping the composition of the gut microbiota [1]. The timing of exposure to food allergens in the maternal and infant diet is usually assessed by identifying the age at which the first solid food is introduced, the feeding pattern, and breastfeeding. There exists disagreement, however, whether the maternal ingestion of allergens during pregnancy and lactation is a risk factor for FA development or other atopic diseases [11]. There is also controversy surrounding the role of breastfeeding on FA development, as previous studies have examined the role of breastfeeding predominantly for the risk of atopic diseases, such as asthma and atopic dermatitis, rather than for FA in particular [12]. For infant exposure, recent findings showed that the early introduction of allergenic foods before six months of age prevents the development of FA [13]. Nutritional factors associated with higher prevalence of FA also include either vitamin D deficiency or excessive vitamin D intake, essential fatty acid deficiencies, and obesity [2]. Studies show that an inadequate vitamin D status, predominantly caused by insufficient sunlight exposure, was associated with a higher risk of asthma and allergies [7]. Vassallo et al. [14] observed that the season of birth is a risk factor for FA as infants born during winter had a higher risk of developing FA. More studies evaluating population-specific nutritional variables associated with early infancy feeding are clearly needed to address environmental variations related to FA.

There is a lack of information regarding the scope of the FA burden among children in Kuwait. Moreover, it also appears that allergy medical specialists doubt the role of early feeding practices during infancy in FA development and management. Therefore, this investigation aimed to evaluate the association between IgE-mediated food allergy and potential risk factors, including child nutritional status, breastfeeding and its duration, the age of first solid food introduction, and vitamin D status during infancy. As a secondary environmental-related factor, we examined whether the child's antibiotic exposure during the first year of life is related to FA development.

## 2. Methods

### 2.1. Study Design

This study was a case-control investigation using FA cases from pediatric allergy and immunology clinics (PAICs) in the main government hospitals in Kuwait from August to December 2015. The controls were additionally recruited between August 2015 and February 2016 from various vaccination units at primary healthcare centers.

### 2.2. Case Ascertainment and Recruitment

For eligibility, we conducted a prospective review of data from pediatric patients registered as case referrals to the outpatient PAICs, located in hospitals under the jurisdiction of the Ministry of Health in Kuwait, including the Al-Amiri, Al-Sabah, Al-Adan, and Mubarak Al-Kabeer Hospitals. We screened a total of 584 children (0–13 years of age) presented with atopic diseases seen consecutively from August to December 2015. The identified patients were invited to participate in the study during a multicenter recruitment process conducted at the outpatient PAICs located in the participating hospitals. The allergy and immunology clinics working hours were only for once a week, and an average of 8–10 children per week were seen at each clinic. Ethical approval for the study was obtained from the Ministry of Health, and informed consent forms were obtained from all the parents of the participating children. Participation was voluntary and did not affect the patients' rights to treat. All participating parents were asked to complete a survey tool designed to collect information on their child's allergies and the associated factors. The survey questionnaire was developed and pretested to collect demographic and early feeding practices and FA-related information. Also, types of food allergens and the presence of a diagnosis other atopic diseases (i.e., eczema, asthma, and rhinitis) were determined. Cases with missing diagnosis and incomplete questionnaires were excluded. In the case of healthy children, those who were attending the vaccination centers and did not have chronic illnesses were included. All children who were reported by their parents as having “ever” had a food allergy and were diagnosed by a physician were excluded.

### 2.3. Definitions

Confirmed FA was defined as having a convincing clinical history of an IgE-mediated reaction attributed to food, confirmed by an allergist, using either a positive skin prick test (SPT) (+3 mm) or specific IgE > 0.35 kU/L. Anaphylaxis was defined as signs and symptoms that developed immediately after exposure to certain foods and involved at least two major organ systems according to established guidelines [10]. The World Health Organization (WHO) standards were used to assess the children's nutritional status [15]. Normal growth was defined as a weight-for-age (WAZ) and height for age (HAZ) of 0 to ±2 *Z*-score, moderate undernutrition –2 to –3 *Z*-score, and severe undernutrition <–3 *Z*-score. Conversely, being overweight was defined as having a WAZ > +2 *Z*-score and obesity as >+3 *Z*-score [16]. A low HAZ (<–2 *Z*-score) indicated stunting, and WAZ <–2 *Z*-score indicated a child just being underweight. For serum vitamin D, a value of 50 nmol/L marks sufficiency in 25(OH) D levels, below 25 nmol/L was considered as deficient, and levels between 25 and 50 nmol/L were considered insufficient [17].

### 2.4. Survey Questionnaire and Study Variables

The FA questionnaire was developed and standardized according to the International Study of Asthma and Allergies in Childhood (ISAAC) 2010 questionnaire. The questionnaire comprised sections on the child's history of allergic symptoms and asthma. Some modifications were made to incorporate questions regarding food allergies and nutritional factors. The initial version was reviewed by two pediatric experts who were consultants that specialized in allergies and clinical immunology. The questionnaire was tested in a pilot study for content and face validity. The final questionnaire consisted of four parts including: (a) general and sociodemographic data; (b) food allergy and child's diet history; (c) maternal information; and (d) specific laboratory tests.

The requested information on FAs included the types of food that caused allergic reactions, symptoms, age at which FA was diagnosed, and anaphylaxis history. The FA-associated factors that were surveyed included the methods of infant feeding, breastfeeding and its duration, age at which infant formula was introduced, type of initial and current formula being used, age of complementary food introduction, intake of child vitamin D supplements, child vitamin D status (existing diagnosed deficiency or insufficiency), and the child's antibiotic exposure in the first year of life.

### 2.5. Statistical Analysis

Descriptive statistics provided the mean and standard deviation in order to describe the continuous quantitative data. A chi-squared test was used to compare the characteristics of the cases and controls and to examine the relationship between FA and categorical variables that included nutritional factors. For the nutritional assessment, *Z*-scores were calculated for WAZ, HAZ, and body mass index (BMI) for age (BAZ); they were based on the WHO reference standard [10]. For each index, we created three categories according to the standard deviation (SD): <–2 = undernourished, 0 to +2 = normal, and >+2 = above normal. Univariate logistic regression analyses were conducted to investigate the FA-associated nutritional factors and to decide on the final variables to be included in the final multivariate modeling. Multivariate logistic regression models were used to determine the independent variables and to identify the potential confounders in the relationships explored. Factors without influence on the exposure or outcome were excluded from the multiple logistic regression models. All statistical tests were two-sided. A *p* value of <0.05 was considered significant. Data were analyzed using WHO AnthroPlus software and the Statistical Package for Social Sciences (version 20.0) for Windows.

## 3. Results

**Figure 1:**
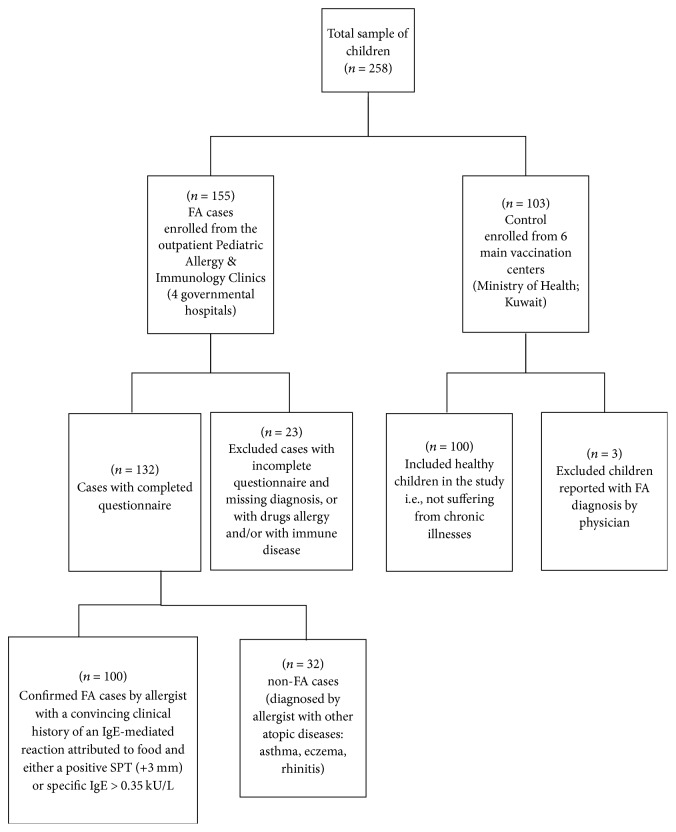
Flow chart of participants' selection.

For the chart review, after the duplicated cases were checked and removed, we identified 433 out of 584 case referrals to the outpatient PAICs. Almost half of the cases (46.9%, *n* = 203) were diagnosed with FAs, whereas 46.7% (*n* = 202) were diagnosed with other types of atopic diseases. The diagnosis was missing for 6.4% (*n* = 28) of the children. During the study period, 29% (*n* = 58) of the FA cases were identified as new case referrals, and 71% (*n* = 144) were follow-up cases. Accordingly, the incidence rate of FA in the PAICs during the period of our study was 14.4% [(58/404) × 100] (Figure 1).

For the case-control study, a total of 155 questionnaires were distributed among FA cases, and 132 were completed. Of the 132 FA cases, 75.8% (*n* = 100) were with “confirmed FA” by an allergist; 24.2% (*n* = 32) were identified as non-FA cases. The prevalence of confirmed FA according to age group was 37% (*n* = 37) among those aged 0–2 years, 39% (*n* = 39) among those aged >2–5 years, and 24% (*n* = 24) among those aged >5–13 years. Children aged 0–5 years displayed a higher prevalence of FA than the other age groups (76%, *n* = 76) (*χ*^2^ = 6.09; *p* = 0.01). We found that younger children were likelier to have FAs (odds ratio [OR] = 2.8; confidence interval [CI] = 1.2–6.4; *p* = 0.016).

The characteristics of the cases and controls in the total study sample and as categorized per age group are summarized in Table 1. There were no statistically significant differences in the sociodemographic information between the cases and controls in the total sample or within each age category. Also, there were no statistically significant differences in the growth parameters between the cases and controls in the total sample [WAZ, *p* = 0.58; HAZ, *p* = 0.23; and BAZ, *p* = 0.24] and within the age categories.

**Figure 2:**
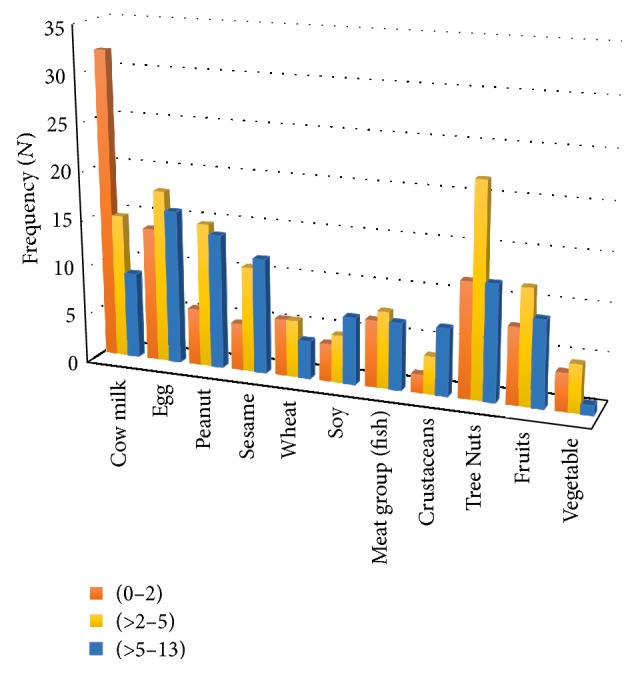
Common food allergens reported in children with confirmed food allergy, stratified by age group, in years (*n* = 100). Note: some cases were reported to have more than one type of food allergen (*n* = 33).

More than 27% of the cases (27.6%, *n* = 24) were allergic to one food; 3.4% (*n* = 3) were allergic to two foods, and 69% (*n* = 60) were allergic to two or more foods. Children who were allergic to >2 foods were found to have significantly lower mean HAZ scores than those allergic to ≤2 foods [mean ± SD, 0.045 ± 2.28 versus 1.18 ± 3.18, *p* = 0.04]. Cow's milk was the most commonly reported food allergen in children aged 0–13 years (56%, *n* = 56), followed by eggs (48%, *n* = 48), tree nuts (46%, *n* = 46), and peanuts (35.4%, *n* = 35) (Figure 2). The types of food allergens differed significantly among the age groups. Allergies to cow's milk and eggs were more common among younger children (0–5 years) than those >5–13 years (*p* = 0.036); crustaceans, as allergens, were more common among those >5–13 years than those aged 0–5 years (*p* = 0.021) (Figure 2).

**Figure 3:**
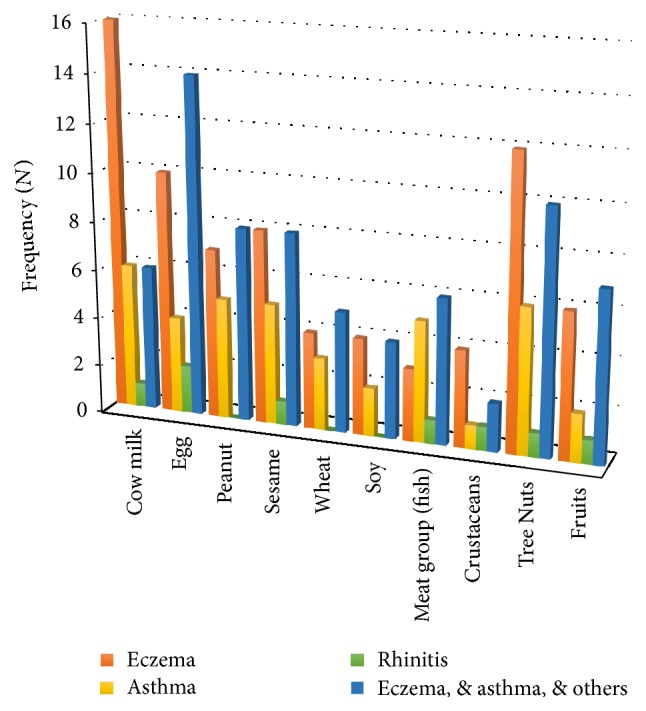
The most common food allergens in children with confirmed food allergy, stratified by type of atopic disease.

FA status was associated with other atopic diseases such as asthma and eczema. Forty-two percent of the FA cases (42.2%, *n* = 21) presented with eczema only, 21.6% (*n* = 11) with asthma only and 5.9% (*n* = 3) with rhinitis only; 31.4% (*n* = 16) had both eczema and asthma with other allergies (Table 2). The cases presenting with eczema in combination with asthma were associated with egg allergy (*χ*^2^ = 8.8; *p* = 0.03). Furthermore, there was a notable association between eczema and cow's milk allergy, as well as asthma and fish allergy (Figure 3). Anaphylactic reactions were reported among 63.9% (*n* = 62) of the FA cases (Table 2). The anaphylaxis-inducing foods reported by parents were cow's milk and milk products (*n* = 27/51), tree nuts (*n* = 11/51), and eggs (*n* = 9/51).

Most of the early feeding practices and exposures were significantly correlated with FA in the total sample and within age categories (Table 3). Compared to controls, FA cases were more likely to be exclusive breastfed (64.9% versus 35.1%, *χ*^2^ = 12.87, *p* < 0.001), whereas FA cases were less likely to be breastfed for the recommended duration, that is, for 6 months or more (48.0% versus 52.0%, *χ*^2^ = 6.37, *p* = 0.012), less likely were given mixed feedings with formula (43.1% versus 56.9%, *χ*^2^ = 8.97, *p* = 0.003), and less likely were introduced to complementary feeding at less than 6 months (47.6% versus 52.5%, *χ*^2^ = 8.05, *p* = 0.005).

Using unadjusted binary logistic regression (Table 4) also showed that a higher odds of FAs was associated with exclusive breastfeeding (OR = 3.01; *p* < 0.001), with a longer breastfeeding duration (≥6 months) (OR 2.67, *p* = 0.012), and with delaying the introduction of complementary-foods in the first year of life (>6 months) (OR = 2.98; *p* = 0.001), whereas a lower odds of FA was associated with mixed feedings (breastfeeding supplemented with formula) (OR = 0.38; *p* = 0.003).

With regard to vitamin D status, the proportion of children with a history of vitamin D deficiency was higher among the cases than the controls (11% versus 2%, *χ*^2^ = 6.6; *p* = 0.01). Children with a history of vitamin D deficiency and those who were prescribed vitamin D supplements during infancy were likelier to have FAs (OR = 6.05; *p* = 0.02 and OR = 4.54; *p* < 0.001), respectively. According to the season of birth, however, there were no significant differences found between the two groups. Additionally, 14.1% (*n* = 19) of FA cases were either diagnosed with or had a history of rickets, 5% (*n* = 10) of FA cases had a history of iron-deficiency anemia, and five cases were diagnosed with failure to thrive, as indicated in their medical files.

Exposure to antibiotics during the first year of life was higher among the cases than the controls, at 59.8% and 34.9%, respectively (*χ*^2^ = 10.32; *p* < 0.001). Children exposed to antibiotics were twice as likely to have FAs as the controls (OR = 2.78; *p* = 0.001). Lastly, lower maternal BMI was associated with a higher odds of FA development (OR = 2.08; *p* = 0.02).

The multivariate logistic regression analysis was performed including all the significant correlates with FA status adjusting for age and maternal BMI (Table 5). The results showed that the independent relationships between FA status and the investigated early exposures included exclusive breastfeeding (aOR = 15.55 (3.26–74.19), *p* = 0.001), vitamin D deficiency or insufficiency during infancy (aOR = 5.42 (1.92–15.30), *p* = 0.001), and antibiotic exposure during the first year of life (aOR = 5.00 (1.58–15.84), *p* = 0.006). This model explained 33.6–45.6% of the variations in FA status.

## 4. Discussion

The present study data showed a high FA burden among children at the PAICs in Kuwait. Almost one-half of the new case referrals to the PAIC (46.9%) had a confirmed FA diagnosis, and the incidence rate was 14.4% during our study period as obtained from the registry records. These findings, however, demonstrate a higher point prevalence rate than observed in similar studies previously conducted in allergy clinics. For example, at the allergy clinic of the King Khalid University Hospital in Riyadh, the prevalence of IgE-specific FA was 32.9% (*n* = 92/280, age < 12 years) [18]. Al-Hammadi et al. [19] reported a lower point prevalence of 17.8% (*n* = 68/386) regarding confirmed FA among children with atopic dermatitis (age range < 1–8 years) seen in multidisciplinary dermatology/pediatric allergy clinics in France. In contrast, a lower prevalence of FA has been noted in the general population as opposed to subjects who visited allergy clinics. For instance, the FA prevalence in Al-Ain city was reported to be 8% among 397 school children aged 6–9 years and 4.6% among 3,827 Chinese preschool children aged 2–7 years in Hong Kong [20, 21]. It is difficult to directly compare the present study findings with data from other studies due to methodological differences and the variations in the age range among studies. The validity of the estimates in the present work is strengthened given that the records of patients were retrieved from multiple sites and used physician-diagnosed criteria.

The most common allergenic foods reported among our sample were cow's milk, eggs, tree nuts, and peanuts. These are consistent with other international reports [22]. It has been observed that the order of the most common food allergens varies by country, reflecting genetic factors, dietary habits, and exposure to allergenic foods early in life [22]. For example, seafood was found to be a common food allergen in Asia, cow's milk in the Middle East, egg and cow's milk in Europe, in addition to specific fruit allergens, and peanuts and other nuts in Australia, Western Europe, and the United States [22]. Because of the globalization and westernization of foods and dietary habits around the world, Kuwait may be facing the same epidemic of FA as in the United States and European countries [23]. Moreover, the differences in the food allergens identified in each study may be reflective of the temporal changes in the eating preferences of the selected population. In the Kingdom of Saudi Arabia (KSA), peanuts were found to be the most common food allergen; milk was the third most common allergen in 2000, while a more recently conducted study reported it to be the most common food allergen (61.9%), followed by eggs (59.7%) and wheat (45.65%) [18].

Our data showed that allergies to peanuts, shellfish, and tree nuts were more common among older children, whereas allergies to cow's milk and eggs were more common among younger children [22, 24], as older children may have outgrown their cow's milk allergy, by the age of 5 years [25]. Similar observations are reported in KSA, where cow's milk allergy was most common among children, while egg allergy was the most common among adults [18]. In Lebanon, cow's milk was found to be the major cause of FA among infants and young children, while hazelnut and wheat flour allergies were the most common among adults [26].

The anaphylaxis-inducing foods identified in our sample were cow's milk and dairy products, tree nuts, and eggs. In Iran, which is also within our region, milk and dairy products were reported to be the most common allergens that induced anaphylaxis among children [27]. In contrast, peanuts are the leading cause of deaths related to food-induced anaphylaxes in the United States [28] whereas in our study peanut allergy was not found as frequently to cause anaphylaxis in children. In Israel, sesame was found to be a major cause of anaphylaxis among children and was second only to cow's milk [29]. The above findings suggest that the observed differences of common anaphylaxis-inducing foods among regions are related to geographical or/and environmental exposures in the communities investigated. Nonetheless, the geographical and environmental impacts require further investigations beyond the scope of this study.

A significant number of FA cases in our sample were diagnosed with eczema and had associated atopic diseases, including asthma and rhinitis. There is a well-documented link between the presence of early eczema in childhood and the development of FAs, especially peanut, egg, and milk allergies. Previously conducted studies have reported that between 33% and 81% of children with infant eczema had an IgE-mediated food allergy [28]. The risk of egg, milk, or peanut allergy development was shown to be approximately twice as high if eczema was present in the first 6 months of life than if it was present in the second 6 months of life [29]. In France, among 362 children with asthma, between the ages of 6 and 18 years, 19.3% (*n* = 70) were diagnosed with FA [30]. In another study, 35.9% of asthmatic children in Semnan (Iran) showed sensitization to at least one of the principal allergenic foods (wheat, rice, peanut, egg, soya, and cow's milk) [31].

We found that exclusive breastfeeding for more than six months was associated independently with higher odds of FA (aOR = 15.55; *p* = 0.001). This finding is not unusual as such observations been noted in larger population-based studies. In Finland, the protective effect of atopic diseases through exclusive breastfeeding was present only when the feeding duration was 4–6 months; extended breastfeeding increased the risk [32]. These apparently contradictory findings can be controversial, particularly as exclusive breastfeeding for 4–6 months is advised by many health authorities [33]. One explanation for these results is that the preventative effect of breastfeeding on FA might be influenced by the breastfeeding practices and not only by the duration [12]. For example, in Kuwait, “exclusive breastfeeding” is interpreted to mean that the infant depends primarily on the mother's milk; however, this does not necessarily mean that children are not given juices or solid food. Nasreddine et al. showed that mothers who reported exclusively breastfeeding also gave their children sweetened water, herbal tea, and fruit juices at around 3 to 5 months of age [34]. The introduction of these items reduces the amount of breastmilk consumption during the day and may produce a confounding effect that negatively influences the protective relationship between breastfeeding and FA. Another confounding influence is that prolonged exclusive breastfeeding (>6 months) may delay weaning into solid foods, which may decrease exposure of the child to allergenic foods that could, in turn, lead to an increased risk of FA development [7]. Recent data in Kuwait showed that the percentage of breastfed babies receiving complementary food at 6–8 months of age had increased from 51.4% in 2010 to 81.8% in 2015 [35]. Similarly, we observed, among our FA cases, that the introduction of solid food was likelier be delayed until 6 months or beyond, among exclusively breastfed children. Collectively, these findings are in agreement with several other studies, which state that the protective effects of breastfeeding might be influenced by the quantity of breastmilk consumed, the frequency of breastfeeding, and the allergenic foods introduced early in life [13]. Based on our findings and these recent observations, it can be hypothesized that early infant feeding practices might explain the increase in FA among children in Kuwait, as there appears to be an increasing trend towards delaying the introduction of solid foods.

Another pertinent issue associated with prolonged breastfeeding is the development of vitamin D deficiency in exclusively breastfed infants [2]. Formula milk is fortified with vitamin D to meet growing infants' needs; however, if an exclusively breastfed infant is not provided with vitamin D supplementation, the development of vitamin D deficiency is likelier. In support of this conjecture, the present data indicated that vitamin D deficiency was associated with an increased odd of FA (aOR = 5.42; *p* = 0.001), which is consistent with the findings of recently conducted epidemiological studies [2]. These findings are in agreement with those observed in the United States and Australia although this relationship has not been consistently observed in other countries [14, 36, 37]. Vitamin D is recognized as an essential regulator of immune response that may modulate the effect of breastfeeding on FA development [38]. Another possible immune-modulating factor is variations in vitamin D content in the mother's milk, which is affected by genetic and environmental factors as well as the mother's dietary intake and maternal allergic status [39].

In our sample, the FA status was found to be associated with some nutritional deficiency syndromes, including vitamin D deficiency, stunting, rickets, failure to thrive, and iron-deficiency anemia. These findings are consistent with those of other studies [4, 40–42]. Iron-deficiency anemia was present in 5% of the children of our study sample, with higher ORs being noted among the FA cases (OR = 5.4; *p* = 0.03). The high prevalence of anemia among children with allergies can be related to the use of systemic immunosuppressant medications and food allergen avoidance, which may directly affect their intake of iron-rich foods such as eggs or fortified cereals [43]. Dietary intervention for FA management should aim to address anemia and both undernutrition and overnutrition [44].

The results of the current study indicate that children who were exposed to antibiotics during the first year of life had an increased odds of FA (aOR = 5.00; *p* = 0.006). It is plausible that the higher antibiotic use among FA cases was due to more illnesses experienced by the children with atopic disease. Based on the hygiene hypothesis, antibiotics can cause alterations in immunity and change the intestinal colonization pattern [45]. Although the microbial composition of the gut microbiota was not assessed in the present work, exposure to antibiotics in infancy, as a proxy indicator of disturbed immunity and gut microbial profiles, was significantly associated with FA status. The high prevalence of exposure to antibiotics during the first year of life among our FA cases and controls may be indicative of potential mishandling or overuse of antibiotics. Towards the prevention of atopic diseases, medical care providers should minimize the use of antibiotics, especially during early life [8, 9, 45].

### 4.1. Strengths and Limitations

A study strength was that subjects were selected from PAICs as this provided the opportunity for the use of objective tests (SPT and/or specific IgE test) to confirm the diagnosis of the reported FA, which is unlikely to be achievable in larger population-based studies. As the study was conducted among a selected population, more information could be obtained regarding FAs and their associations with the presence of atopic diseases. Although there were differences in the age groups between the cases and controls due to time limitations and recruitment restrictions, the age variation was adjusted by converting anthropometric measurements into *z*-scores and a standardized age- and sex-specific growth reference, based on the WHO reference standard. The number of participants with FA was small (*n* = 100); therefore, detailed analysis regarding onset and duration per allergenic food were not possible. Also, for the vitamin D status during infancy, retrospective data from chart reviews were used; thus the onset of vitamin D deficiency could not be determined. The possibility of recall bias in the study population could not be ruled out as participants self-selected themselves to the study as participation was not mandatory.

## 5. Conclusions

A high prevalence of FA was observed in the children in the present study. Inappropriate dietary practices during infancy, including the delayed introduction of complementary feeding and exclusive breastfeeding for more than six months, vitamin D deficiency during infancy, and more frequent use of antibiotics during the first year of life, were associated with FA development. Cow's milk, eggs, and tree nuts were the most common allergens among children with FA and were associated with atopic diseases such as asthma and eczema. FA was also associated with nutritional deficiency syndromes, including rickets, failure to thrive, and iron-deficiency anemia.

## Figures and Tables

**Table 1:** Characteristics in FA cases versus controls in the total sample and within age categories.

Characteristics	Categories	Total (*n* = 200)		Age Categories, *n* (%)					
				Less than 2 years old, 105 (52.5)		2–5 years old, 65 (32.5)		>5 years old, 30 (15)	
		FA *n* = 100	Control *n* = 100	FA 37 (35.2)	Control 68 (64.8)	FA 39 (60)	Control 26 (40)	FA 24 (80)	Control 6 (20)
Gender	Male	67 (54.9)	55 (45.1)	23 (36.5)	40 (63.5)	26 (68.4)	12 (31.6)	18 (85.7)	3 (14.3)
	Female	33 (42.3)	45 (57.7)	14 (33.3)	28 (66.7)	13 (48.1)	14 (51.9)	6 (66.7)	3 (33.3)

Nationality	Kuwaiti	48 (45.7)	57 (54.3)	19 (32.8)	39 (67.2)	17 (53.1)	15 (46.9)	12 (80)	3 (20)
	Expats	52 (54.7)	43 (4.3)	18 (38.3)	29 (61.7)	22 (66.7)	11 (33.3)	12 (80)	3 (20)

Season of Birth	Dec-Feb	28 (63.4)	16 (36.4)	9 (45.0)	11 (55.0)	11 (84.6)	2 (15.4)	8 (72.7)	3 (27.3)
	Mar-May	23 (52.3)	21 (47.7)	13 (52)	12 (48)	6 (42.9)	8 (57.1)	4 (80)	1 (20)
	Jun-Aug	33 (45.8)	39 (54.2)	11 (27.5)	29 (72.5)	14 (60.9)	9 (39.1)	8 (88.9)	1 (11.1)
	Sept-Nov	16 (41)	23 (59)	4 (20.0)	16 (80.0)	8 (57.1)	6 (42.9)	4 (80)	1 (20)

Gestational age	Full-term	85 (52.2)	77 (47.5)	29 (33.7)	57 (66.3)	29 (55.8)	23 (44.2)	19 (79.2)	5 (20.8)
	Preterm	14 (70)	6 (30)	4 (40.0)	6 (60.0)	8 (100)	0	2 (100)	0

Family income	≤1000 KD	37 (49.3)	38 (50.7)	12 (31.6)	26 (68.4)	15 (62.5)	9 (37.5)	10 (76.9)	3 (23.1)
	>1000 KD	55 (47.8)	60 (52.2)	19 (32.2)	40 (67.8)	22 (56.4)	17 (43.6)	14 (82.4)	3 (17.6)

Maternal age	<25 yrs	20 (46.5)	23 (53.5)	5 (26.3)	14 (73.7)	11 (64.7)	6 (35.3)	4 (57.1)	3 (42.9)
	≥25 yrs	78 (50.3)	77 (49.7)	32 (37.2)	54 (62.8)	28 (58.3)	20 (41.7)	18 (85.7)	3 (14.3)

Maternal BMI	Underweight	6 (60)	4 (40)	1 (20)	4 (80)	3 (100)	0	2 (100)	0
	Normal	46 (54.8)	38 (45.2)	15 (36.6)	26 (63.4)	21 (70)	9 (30)	10 (76.9)	3 (23.1)
	≥Overweight	30 (38.5)	48 (61.5)	16 (34.8)	30 (65.2)	9 (37.5)_a_	15 (62.5)_b_	5 (62.5)	3 (37.5)

Birth Weight	<2.5 kg	13 (59.1)	9 (40.9)	4 (33.3)	8 (66.7)	7 (100)	0	2 (66.7)	1 (33.3)
	=>2.5 kg	76 (45.5)	91 (54.4)	30 (33.3)	60 (66.7)	28 (51.9)	26 (48.1)	18 (78.3)	5 (21.7)

Weight for age, *Z-scores*	Thinness, <−2SD	5 (50)	5 (50)	2 (40)	3 (60)	2 (50)	2 (50)	1 (100)	0
	Normal, −2 to +2SD	75 (46.9)	85 (53.1)	26 (30.6)	59 (69.4)	33 (60)	22 (40)	16 (80)	4 (20)
	Overweight, >+2SD	14 (58.3)	10 (41.7)	9 (60)	6 (40)	4 (66.7)	2 (33.3)	1 (33.3)	2 (66.7)

Length or stature for age, *Z-scores*	Low, <−2SD	11 (64.7)	6 (35.3)	2 (33.3)	4 (66.7)	8 (80)	2 (20)	1 (100)	0
	Normal, >+2SD	71 (46.7)	81 (53.3)	20 (25.6)	58 (74.4)	29 (61.7)	18 (38.3)	22 (81.5)	5 (18.5)
	High, >+2SD	18 (58.1)	13 (41.9)	15 (71.4)	6 (28.6)	2 (25)	6 (75)	1 (50)	1 (50)

BMI for age, *Z-scores*	Thinness, <−2SD	8 (42.1)	11 (57.9)	6 (54.5)	5 (45.5)	2 (28.6)	5 (71.4)	0	1
	Normal, −2 to +2SD	75 (48.4)	89 (51.6)	26 (31.3)	57 (68.7)	28 (59.6)	19 (40.4)	21 (84)	4 (16)
	Overweight, >+2SD	17 (65.4)	9 (34.6)	5 (45.5)	6 (54.5)	9 (81.8)	2 (18.2)	3 (75)	1 (25)

Values are presented as number, *n*, and percent (%). Each subscript letter denotes a subset of FA cases versus controls characteristics categories whose column proportions do not differ significantly from each other at 0.05 level; % characteristics categories compared between FA cases and controls. *Chi-square statistics are as follows:* for the gestational age [2–5-year age category], *χ*^2^ = 5.74 (1), *p* = 0.017; maternal BMI [2–5-year age category], *χ*^2^ = 8.08 (2), *p* = 0.018.

**Table 2:** Prevalence of other atopic diseases among confirmed^a^ FA cases.

Physician diagnosed Cases (*n* = 51)	*n* (%)
Eczema only	21 (42.2)
Asthma only	11 (21.6)
Rhinitis only	3 (5.9)
Eczema, asthma, and others	16 (31.4)

Parents- reported symptoms in children with food allergy^b^ (*n* = 100)	*n* (%)

Skin	87 (88.8)
Respiratory	44 (44.9)
Gastrointestinal	43 (43.9)
Anaphylaxis^c^	62 (63.9)
One time	12 (19.4)
2-3 times	19 (30.6)
>3 times	31 (50)

^a^Confirmed food allergy by an allergist and through tests (skin prick test and/or sIgE test). ^b^Parents'-reported symptoms. Skin: rash, itching, and urticaria. Respiratory: coughing, sneezing, and difficulty in breathing. Gastrointestinal: abdominal pain, nausea and vomiting, and diarrhea. ^c^Anaphylaxis: anaphylaxis was defined as the rapid development of symptoms after exposure that affected at least two major organs [10].

**Table 3:** Comparison of early feeding practices and exposures between FA cases and controls in the total sample and within age categories.

Characteristics	Total Sample (*n* = 200)		Age Categories, *n* (%)					
			<2 years old, 105 (52.5)		2–5 years old, 65 (32.5)		≥5 years old, 30 (15)	
	FA 100 (50)	Control 100 (50)	FA 37 (35.2)	Control, 68 (64.8)	FA 39 (60)	Control 26 (40)	FA 24 (80)	Control 6 (20)
Breastfed ever								
Yes	83 (52.2)	76 (47.8)	26 (33.8)	51 (66.2)	36 (64.3)	20 (35.7)	21 (80.8)	5 (19.2)
No	17 (44.7)	21 (55.3)	11 (42.3)	15 (57.7)	3 (37.5)	5 (62.5)	3 (75)	1 (25)
Exclusive breastfeeding for six months or more								
Yes	48 (64.9)_a_	26 (35.1)_b_	16 (45.7)_a_	19 (54.3)_b_	22 (78.6)_a_	6 (21.4)_b_	10 (90.9)	1 (9.1)
No	43 (38.1)_a_	70 (61.9)_b_	15 (24.2)_a_	47 (75.8)_b_	16 (45.7)_a_	19 (54.3)_b_	12 (75)	4 (25)
Breastfeeding duration*, in months*								
<6	18 (25.7)_a_	52 (74.3)_b_	8 (18.2)	36 (81.8)	5 (31.3)	11 (68.8)	5 (50)	5 (50)
≥6	24 (48)_a_	26 (52)_b_	5 (23.8)	16 (76.2)	15 (62.5)	9 (37.5)	4 (80)	1 (20)
Supplemented with formula								
Yes	59 (43.1)_a_	78 (56.9)_b_	24 (31.6)	52 (68.4)	20 (50)_a_	20 (20)_b_	15 (71.4)	6 (28.6)
No	38 (66.7)_a_	19 (33.3)_b_	12 (46.2)	14 (53.8)	17 (77.3)_a_	5 (22.7)_b_	9 (100)	0
Introduction of complementary feedings, *age in months*								
<6	59 (47.6)_a_	65 (52.4)_b_	22 (35.5)	40 (64.5)	20 (51.3)_a_	19 (79.2)_b_	17 (73.9)	6 (26.1)
≥6	35 (71.4)_a_	14 (28.6)_b_	10 (52.6)	9 (47.4)	19 (48.7)_a_	5 (20.8)_b_	6 (100)	0
Antibiotics exposure in the first year								
Yes	73 (59.8)_a_	49 (40.2)_b_	27 (42.9)	36 (57.1)	27 (71.1)_a_	11 (28.9)_b_	19 (90.5)_a_	2 (9.5)_b_
No	22 (34.9)_a_	6 (21.4)_b_	7 (23.3)	23 (76.7)	12 (46.2)_a_	14 (53.8)_b_	3 (42.9)_a_	4 (57.1)_b_
Vitamin D deficient or insufficient								
Yes	36 (56.3)_a_	28 (43.8)_b_	16 (45.7)_a_	19 (54.3)_b_	16 (66.7)_a_	8 (33.3)_b_	4 (80)	1 (20)
No	15 (22.1)_a_	53 (77.9)_b_	6 (15.8)_a_	32 (84.2)_b_	6 (27.3)_a_	16 (72.7)_b_	3 (37.5)	5 (62.5)

Values are presented as number, *n*, and percent (%). Each subscript letter denotes a subset of FA cases versus controls characteristics categories whose column proportions do not differ significantly from each other at 0.05 level; % characteristics categories compared between FA cases and controls. *Chi-square (χ*^2^*) statistics are as follows:* for the *ever* breastfed [total], *χ*^2^ = 12.87 (1), *p* < 0.001; [<2-year age category], *χ*^2^ = 4.77 (1), *p* = 0.029; [2–5-year age category], *χ*^2^ = 7.02 (1), *p* = 0.008; breastfeeding duration [total], *χ*^2^ = 6.37 (1), *p* = 0.012; supplemented with formula [total], *χ*^2^ = 8.97 (1), *p* = 0.003; [2–5-year age category], *χ*^2^ = 4.39 (1), *p* = 0.036; introduction of complementary feeding [total], *χ*^2^ = 8.05 (1), *p* = 0.005; [2–5-year age category], *χ*^2^ = 4.89 (1), *p* = 0.027; antibiotic exposure [total], *χ*^2^ = 10.32 (1), *p* = 0.001, [2–5-year age category], *χ*^2^ = 4.021 (1), *p* = 0.045; [5–13-year age category], *χ*^2^ = 7.07 (1), *p* = 0.0008; vitamin D deficient or insufficient [total], *χ*^2^ = 16.26 (1), *p* < 0.001; [<2-year age category], *χ*^2^ = 7.75 (1), *p* = 0.005; [2–5-year age category], *χ*^2^ = 7.14 (1), *p* = 0.008.

**Table 4:** Unadjusted odds ratios using univariate logistic regression for FA allergy status as the dependent variable and early feeding practices and exposures as independent variables.

Characteristics	Categories	Total Sample	Age Categories		
			Birth to 2 years	2–5 years	>5 years
		OR (95% CI) *p*	OR (95% CI) *p*	OR (95% CI) *p*	OR (95% CI) *p*
Maternal BMI	Non-overweight	2.08 (1.11–3.91) 0.022	1.0 (0.42–2.36) -	0.20 (0.06–0.63) 0.005	1.05 (0.19–5.76) 0.96
	Overweight & obese	1	1	1	1

Maternal age	<25 years old	0.76 (0.36–1.61) 0.48	1.66 (0.546–5.039) 0.37	0.80 (0.25–2.55) 0.71	0.56 (0.09–3.45) 0.53
	25 years old and above	1	1	1	1

Birth weight	<2.5 kg	1.70 (0.66–4.43) 0.28	-	1.89 (1.47–2.44) 0.02	3.375 (0.53–21.42) 0.18
	≥2.5 kg	1	-	1	1

Breastfed ever	Yes	1.35 (0.66–2.75) 0.41	0.70 (0.280–1.727) 0.43	3.158 (0.68–14.66) 0.13	1.50 (0.12–19.64) 0.76
	No	1	1	1	1

Exclusive breastfeeding for six months or more	Yes	3.01 (1.63–5.53) <0.001	2.94 (1.09–6.38) 0.03	4.13 (1.34–12.72) 0.01	1.17 (0.10–13.32) 0.90
	No	1	1	1	1

Breastfeeding duration	≥6 months	2.67 (1.23–5.76) 0.012	1.406 (0.39–4.97) 0.60	4.13 (1.06–16.10) 0.04	4.17 (0.42–41.76) 0.20
	<6 months	1	1	1	1

Supplemented with formula	Yes	0.378 (0.20–0.72) 0.003	0.538 (0.22–1.34) 0.179	0.310 (0.095–1.006) 0.046	2.0 (0.24–16.36) 0.515
	No	1	1	1	1

Introduction of complementary feedings	≥6 months	2.98 (1.52–5.84) 0.001	2.26 (0.82–6.25) 0.111	29.23 (3.41–250.92) <0.001	1.47 (1.10–1.95) 0.05
	<6 months	1	1	1	1

Antibiotics exposure in the first year	Yes	2.78 (1.48–5.22) 0.001	2.46 (0.92–6.58) 0.07	2.66 (0.93–7.62) 0.065	2.73 (0.44–17.05) 0.27
	No	1	1	1	1

Vitamin D deficient or insufficient	Yes	4.54 (2.13–9.68) <0.001	4.49 (1.50–13.45) 0.005	6.10 (1.68–22.19) 0.005	15.83 (2.05–122.07) 0.003
	No	1	1	1	1

Values are presented as the unadjusted odds ratio (OR) and 95% confidence interval (95%) CI, followed by the *p* = value (*p*). <0.05 is considered statistically significant.

**Table 5:** Multivariate logistic regression analysis to assess independent relationships between FA status and early exposures during infancy in children.

Variable	aOR	95% CI	*p*-Value
Exclusive breastfeeding for six months or more	15.55	3.26–74.19	0.001
Supplemented with formula	3.53	0.78–16.08	0.103
Introduction of complementary feedings, age in months	2.62	0.85–8.08	0.093
Vitamin D status deficient or insufficient	5.42	1.92–15.30	0.001
Antibiotics exposure in the first year	5.00	1.58–15.84	0.006
Maternal BMI	0.841	0.23–3.03	0.791
Child's age	1.21	0.96–1.51	0.104

aOR, adjusted odds ratio; CI, confidence interval; *p* < 0.05 is considered statistically significant.

## Data Availability

Data are available from the corresponding author upon reasonable request.

## References

[1] Ciardiello M. A., Tamburrini M., Liso M., Crescenzo R., Rafaiani C., Mari A. (2013). Food allergen profiling: A big challenge. *Food Research International*.

[2] Sicherer S. H., Sampson H. A. (2014). Food allergy: epidemiology, pathogenesis, diagnosis, and treatment. *The Journal of Allergy and Clinical Immunology*.

[3] Wang J., Sampson H. A. (2011). Food allergy. *The Journal of Clinical Investigation*.

[4] Giovannini M., D'Auria E., Caffarelli C. (2014). Nutritional management and follow up of infants and children with food allergy: Italian Society of Pediatric Nutrition/Italian Society of Pediatric Allergy and Immunology Task Force Position Statement. *Italian Journal of Pediatrics*.

[5] Cho H.-N., Hong S., Lee S.-H., Yum H.-Y. (2010). Nutritional status according to sensitized food allergens in children with atopic dermatitis. *Allergy, Asthma & Immunology Research*.

[6] Hong X., Wang X. (2012). Early life precursors, epigenetics, and the development of food allergy. *Seminars in Immunopathology*.

[7] Lack G. (2012). Update on risk factors for food allergy. *The Journal of Allergy and Clinical Immunology*.

[8] Hirsch A. G., Pollak J., Glass T. A. (2017). Early-life antibiotic use and subsequent diagnosis of food allergy and allergic diseases. *Clinical & Experimental Allergy*.

[9] Love B. L., Mann J. R., Hardin J. W., Lu Z. K., Cox C., Amrol D. J. (2016). Antibiotic prescription and food allergy in young children. *Allergy, Asthma & Clinical Immunology*.

[10] Sampson H. A., Munoz-Furlong A., Campbell RL., Adkinson N. F., Bock S. A., Branum A. (2006). Second symposium on the definition and management of anaphylaxis: summary report- Second National institute of allergy and infectious disease/food allergy and anaphylaxis network symposium. *Journal of Allergy and Clinical Immunology*.

[11] Katz Y., Rajuan N., Goldberg M. R. (2010). Early exposure to cow's milk protein is protective against IgE-mediated cow's milk protein allergy. *The Journal of Allergy and Clinical Immunology*.

[12] Greer F. R., Sicherer S. H., Burks A. W. (2008). Effects of early nutritional interventions on the development of atopic disease in infants and children: The role of maternal dietary restriction, breastfeeding, timing of introduction of complementary foods, and hydrolyzed formulas. *Pediatrics*.

[13] Perkin M. R., Logan K., Marrs T. (2016). Enquiring about Tolerance (EAT) study: Feasibility of an early allergenic food introduction regimen. *The Journal of Allergy and Clinical Immunology*.

[14] Vassallo M. F., Banerji A., Rudders S. A., Clark S., Mullins R. J., Camargo C. A. (2010). Season of birth and food allergy in children. *Annals of Allergy, Asthma & Immunology*.

[15] World Health Organization (2012). *Global Database on Child Growth and Malnutrition: Child Growth Indicators and their Interpretation*.

[16] World Health Organization & UNICEF (2009). *WHO Child Growth Standards and the Identification of Severe Acute Malnutrition in Infants and Children*.

[17] Shakoor Z., A M., S N. (2016). Screening for food specific ige antibodies among saudi patients with clinical suspicion of food allergy. *International Journal of Advanced Research*.

[18] Mailhol C., Giordano-Labadie F., Lauwers-Cances V., Ammoury A., Paul C., Rance F. (2014). Point prevalence and risk factors for food allergy in a cohort of 386 children with atopic dermatitis attending a multidisciplinary dermatology/paediatric allergy clinic. *European Journal of Dermatology*.

[19] Al-Hammadi S., Al-Maskari F., Bernsen R. (2010). Prevalence of food allergy among children in Al-Ain City, United Arab Emirates. *International Archives of Allergy and Immunology*.

[20] Leung T. F., Yung E., Wong Y. S., Lam C. W. K., Wong G. W. K. (2009). Parent-reported adverse food reactions in Hong Kong Chinese pre-schoolers: Epidemiology, clinical spectrum and risk factors. *Pediatric Allergy and Immunology*.

[21] Prescott S. L., Pawankar R., Allen K. J. (2013). A global survey of changing patterns of food allergy burden in children. *World Allergy Organization Journal*.

[22] Jackson K. D., Howie L. D., Akinbami L. J. (2013). Trends in allergic conditions among children: United States, 1997-2011.. *NCHS Data Brief*.

[23] Rona R. J., Keil T., Summers C. (2007). The prevalence of food allergy: a meta-analysis. *The Journal of Allergy and Clinical Immunology*.

[24] Han Y., Kim J., Ahn K. (2012). Food allergy. *Korean Journal of Pediatrics*.

[25] Irani C., Maalouly G., Germanos M., Kazma H. (2011). Food allergy in Lebanon: is sesame seed the ‘Middle Eastern’ peanut. *World Allergy Organization Journal*.

[26] Teymourpour P., Pourpak Z., Fazlollahi MR. (2012). Cows milk anaphylaxis in children first report of Iranian Food Allergy Registry. *Iran J Allergy Asthma Immunol11*.

[27] Bock S. A., Muoz-Furlong A., Sampson H. A. (2001). Fatalities due to anaphylactic reactions to foods. *The Journal of Allergy and Clinical Immunology*.

[28] Dalal I., Binson I., Reifen R. (2002). Food allergy is a matter of geography after all: sesame as a major cause of severe IgE-mediated food allergic reactions among infants and young children in Israel. *Allergy*.

[29] Hill D. J., Hosking C. S., de Benedictis F. M., Oranje A. P., Diepgen T. L., Bauchau V. (2007). Confirmation of the association between high levels of immunoglobulin E food sensitization and eczema in infancy: an international study. *Clinical & Experimental Allergy*.

[30] Krogulska A., Białek J., Funkowicz M., Wąsowska-Królikowska K. (2013). The prevalence of IgE-dependent food allergy in asthmatic children. *Clinical and Translational Allergy*.

[31] Nabavi M., Hoseinzadeh Y., Ghorbani R., Nabavi M. (2010). Prevalence of food allergy in asthmatic children under 18 years of age in Semnan-Iran in 2007-2008. *Koomesh*.

[32] Hatakka K., Piirainen L., Pohjavuori S., Poussa T., Savilahti E., Korpela R. (2009). Allergy in day care children: Prevalence and environmental risk factors. *Acta Paediatrica*.

[33] Fleischer D. M., Sicherer S., Greenhawt M. (2015). Consensus communication on early peanut introduction and the prevention of peanut allergy in high-risk infants. *Allergy, Asthma & Clinical Immunology*.

[34] Nasreddine L., Zeidan M. N., Naja F., Hwalla N. (2012). Complementary feeding in the MENA region: Practices and challenges. *Nutrition, Metabolism & Cardiovascular Diseases*.

[35] Kuwait Ministry of Health and World Breastfeeding Trends Initiative (2015). Kuwait WBTi Report 2015. *Kuwait Breastfeeding Promotion and BFHI Implementation Committee in collaboration with International Baby Food Action Network (IBFAN) Asia*.

[36] Mullins R. J., Clark S., Katelaris C., Smith V., Solley G., Camargo C. A. (2011). Season of birth and childhood food allergy in Australia. *Pediatric Allergy and Immunology*.

[37] Hoxha M., Zoto M., Deda L., Vyshka G. (2014). Vitamin D and Its Role as a Protective Factor in Allergy. *International Scholarly Research Notices*.

[38] Vuillermin P. J., Ponsonby A.-L., Kemp A. S., Allen K. J. (2013). Potential links between the emerging risk factors for food allergy and vitamin D status. *Clinical & Experimental Allergy*.

[39] Snijders B. E. P., Damoiseaux J. G. M. C., Penders J. (2006). Cytokines and soluble CD14 in breast milk in relation with atopic manifestations in mother and infant (KOALA Study). *Clinical & Experimental Allergy*.

[40] Fox A. T., Du Toit G., Lang A., Lack G. (2004). Food allergy as a risk factor for nutritional rickets. *Pediatric Allergy and Immunology*.

[41] Yu J. W., Pekeles G., Legault L., McCusker C. T. (2006). Milk allergy and vitamin D deficiency rickets: A common disorder associated with an uncommon disease. *Annals of Allergy, Asthma & Immunology*.

[42] Vieira M. C., Morais M. B., Spolidoro J. V. N. (2010). A survey on clinical presentation and nutritional status of infants with suspected cow' milk allergy. *BMC Pediatrics*.

[43] Drury K. E., Schaeffer M., Silverberg J. I. (2016). Association between Atopic Disease and Anemia in US Children. *JAMA Pediatrics*.

[44] Meyer R., De Koker C., Dziubak R. (2014). Malnutrition in children with food allergies in the UK. *Journal of Human Nutrition and Dietetics*.

[45] Lack G. (2008). Epidemiologic risks for food allergy. *The Journal of Allergy and Clinical Immunology*.

